# An Online Evaluation Method for Random Number Entropy Sources Based on Time-Frequency Feature Fusion

**DOI:** 10.3390/e27020136

**Published:** 2025-01-27

**Authors:** Qian Sun, Kainan Ma, Yiheng Zhou, Zhaoyuxuan Wang, Chaoxing You, Ming Liu

**Affiliations:** 1 Institute of Semiconductors, Chinese Academy of Sciences, Beijing 100083, China; sunqian@semi.ac.cn (Q.S.); makainan@semi.ac.cn (K.M.); zhouyiheng@semi.ac.cn (Y.Z.); wangzhaoyuxuan@semi.ac.cn (Z.W.); youzhaoxing@semi.ac.cn (C.Y.); 2 University of Chinese Academy of Sciences, Beijing 100049, China

**Keywords:** information security, entropy source evaluation, random number generators, time-frequency feature fusion

## Abstract

Traditional entropy source evaluation methods rely on statistical analysis and are hard to deploy on-chip or online. However, online detection of entropy source quality is necessary in some applications with high encryption levels. To address these issues, our experimental results demonstrate a significant negative correlation between minimum entropy values and prediction accuracy, with a Pearson correlation coefficient of −0.925 (*p*-value = 1.07 × 10^−7^). This finding offers a novel approach for assessing entropy source quality, achieving an accurate rate in predicting the next bit of a random sequence using neural networks. To further improve prediction capabilities, we also propose a novel deep learning architecture, Fast Fourier Transform-Attention Mechanism-Long Short-Term Memory Network (FFT-ATT-LSTM), that integrates a simplified soft attention mechanism with Fast Fourier Transform (FFT), enabling effective fusion of time-domain and frequency-domain features. The FFT-ATT-LSTM improves prediction accuracy by 4.46% and 8% over baseline networks when predicting random numbers. Additionally, FFT-ATT-LSTM maintains a compact parameter size of 33.90 KB, significantly smaller than Temporal Convolutional Networks (TCN) at 41.51 KB and Transformers at 61.51 KB, while retaining comparable prediction performance. This optimal balance between accuracy and resource efficiency makes FFT-ATT-LSTM suitable for online deployment, demonstrating considerable application potential.

## 1. Introduction

Random number generators (RNGs) are integral to applications in cryptography and secure communications [1]. These generators are typically classified into two categories: pseudo-random number generators (PRNGs), which produce pseudo-random numbers using deterministic algorithms, and true random number generators (TRNGs), which derive randomness from non-deterministic physical processes. While PRNGs rely on a fixed algorithm and seed to generate pseudo-random numbers with favorable statistical properties, their vulnerability arises from the potential exposure of the algorithm and seed, which may lead to security risks and information leakage [2,3,4,5]. In contrast, TRNGs generate random numbers based on non-deterministic factors such as thermal noise, quantum fluctuations, or chaotic circuit behaviors [6,7,8]. Although these random numbers are theoretically unpredictable, they are susceptible to interference from environmental noise and external attacks, which can degrade their quality and increase security vulnerabilities [9,10]. Therefore, evaluating the security of RNGs is paramount in ensuring their reliability in cryptographic applications.

The randomness of generated numbers is primarily characterized by two factors: statistical properties and unpredictability. Statistical testing is often conducted using established international test suites, such as NIST SP 800-22 [11], AIS 31 [12], Diehard [13], and TestU01 [14], which are effective at detecting statistical flaws in random number sequences. However, while PRNGs may pass these statistical tests, subtle correlations between sequences could still exist, reducing their unpredictability and introducing potential security risks. Entropy is commonly used as a metric to assess unpredictability; however, the methods for calculating entropy vary depending on the underlying entropy source, which restricts their general applicability and flexibility. Consequently, further research and optimization of random number security evaluation methods are needed.

In recent years, deep learning techniques have made significant strides in time series prediction [15], and the quality and correlation of random numbers—considered a specific form of time series data—can be effectively evaluated by predicting the probability of subsequent values. The Feedforward Neural Network (FNN) architecture has proven effective in modeling the inherent relationships within data, enabling the detection of pseudo-random sequences [16,17,18]. The Broad Learning System (BLS), proposed by Chen et al. (2018) [19], has gained widespread application in time series prediction and classification tasks due to its incremental structure and rapid convergence [20,21,22]. Wen et al. [23] employed Long Short-Term Memory (LSTM) networks to assess the randomness of a novel type of DRNG that integrates conventional DRNGs with Physical Unclonable Functions (PUFs). Cai et al. [24] combined LSTM with Temporal Pattern Attention (TPA) for evaluating quantum random numbers. Truong et al. [25] developed a model based on Recurrent Convolutional Neural Networks (RCNN) to detect deterministic noise correlations in quantum random number generators. Li et al. [26] used a Transformer-based approach for predicting quantum random numbers. Recent studies indicate that deep neural networks with integrated attention mechanisms outperform traditional models in time series prediction tasks, offering improved accuracy [27,28,29]. Despite the impressive performance of deep learning in random sequence evaluation, many models remain structurally complex and resource-intensive, with large parameter sizes that complicate hardware implementation and hinder online deployment.

This paper proposes an innovative deep learning framework that enhances the model’s ability to identify hidden correlations within random number sequences by integrating an improved soft attention mechanism with frequency-domain analysis techniques. The framework performs entropy source quality detection indirectly by predicting the accuracy of the entropy source, which not only captures the statistical properties of the random number sequences effectively but also reveals subtle dependencies between these sequences. Experimental results show a significant negative correlation between the minimum entropy and prediction accuracy, with a Pearson correlation coefficient of −0.92. Additionally, this method demonstrates higher prediction accuracy across datasets with different randomness levels, with an accuracy improvement of up to 8% compared to baseline networks. This finding validates the model’s efficiency in revealing hidden dependencies and has significant practical value, particularly in the security detection of on-chip random number generators. Notably, due to its low hardware resource requirements, the model is well-suited for resource-constrained environments, offering a practical and cost-effective solution for random number security analysis.

The structure of this paper is organized as follows: Section 2 introduces true random number generators based on ring oscillators (ROs-RNG) and pseudo-random number generators based on linear congruential methods (LC-RNG), which are used to simulate random number datasets with varying degrees of randomness. It also discusses the security challenges associated with ring oscillator-based RNGs and quantifies the relationship between minimum entropy and prediction accuracy using Pearson correlation coefficients, thereby validating the effectiveness of deep learning in random number quality assessment. Section 3 outlines the neural network model proposed in this study, detailing the data collection and preprocessing methods, as well as the setting of thresholds and evaluation of prediction accuracy. Section 4 compares the proposed method with existing models in terms of prediction accuracy and hardware complexity, highlighting the advantages and practicality of the proposed approach. Additionally, the model is embedded and implemented, demonstrating the feasibility of the algorithm for real-time deployment. Finally, Section 5 presents the conclusions of this study and proposes directions for future research.

## 2. Experimental Preparation

### 2.1. Theoretical Basis Verification

**Figure 1:**
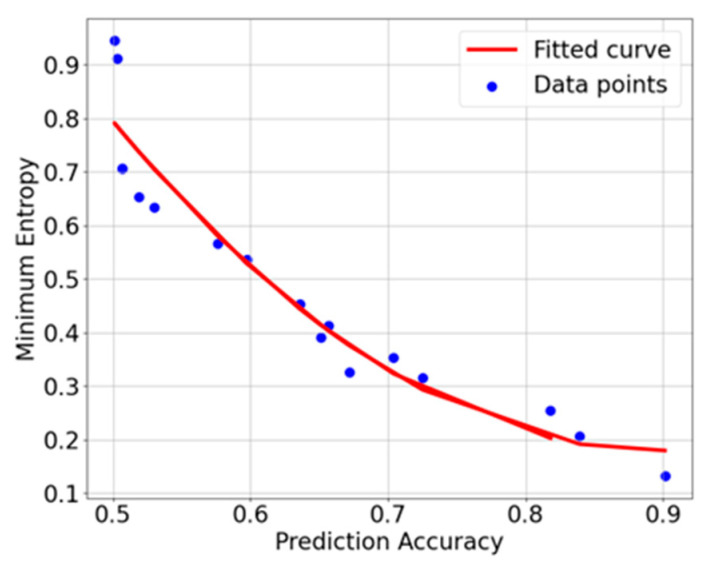
Fitting curve and data points between prediction accuracy and minimum entropy value based on FFT-ATT-LSTM.

Entropy serves as a measure of randomness in random numbers, with better randomness corresponding to an entropy value closer to 1. For random numbers, higher randomness implies that the prediction accuracy should approach 0.5, whereas a higher accuracy indicates some correlation within the sequence. The minimum entropy represents the strictest case of data entropy, and its expression is:

(1) $$H_{min}=-{log}_{2}\underset{1\leq i\leq k}{\max}p_{i}=-{log}_{2}P_{b}$$

where $p_{i}$ represents the probability of 0 or 1 occurring at the i-th bit, and $P_{b}\;$ is the maximum probability of 0 or 1 occurring among the 0 to k bits. The test suite includes multiple predictors, and the one with the highest prediction success rate is selected as $P_{b}$, which is then used to calculate the minimum entropy value. According to the formula for minimum entropy, there exists a negative correlation between minimum entropy and prediction accuracy. To explore the relationship between minimum entropy and prediction accuracy, this paper employs the FFT-ATT-LSTM network to experimentally evaluate random numbers with varying degrees of randomness. The experimental results are shown in Figure 1.

Figure 1 illustrates the correlation between minimum entropy and prediction accuracy. To quantitatively analyze this relationship, the Pearson correlation coefficient is introduced to measure the linear correlation between the two variables. The calculation yields a Pearson correlation coefficient of −0.925 (*p* = 1.07 × 10^−7^, *p* < 0.05), which indicates a significant negative correlation between minimum entropy and the neural network’s prediction accuracy. A *p*-value less than 0.05 suggests that the probability of observing such a strong correlation, assuming no correlation exists, is less than 5%. Therefore, the null hypothesis can be rejected, and the correlation is statistically significant. Based on these findings, the neural network model proposed in this paper can indirectly assess the entropy source quality by predicting the randomness of different random number sequences.

### 2.2. Dataset Preparation

#### 2.2.1. ROs-TRNG Setup

The true random number generator (TRNG) based on multi-ring oscillators is widely utilized in industrial applications due to its distinct entropy source, simple architecture, and ease of implementation [30]. However, phase-locking phenomena may occur during operation [31], which can reduce the number of effective oscillators, thereby diminishing the randomness of the generator’s output. Additionally, environmental factors such as temperature variations and voltage fluctuations can further compromise the randomness, potentially introducing security vulnerabilities. Consequently, evaluating the entropy source quality of multi-ring oscillator-based TRNGs has become a critical and timely research area.

**Figure 2:**
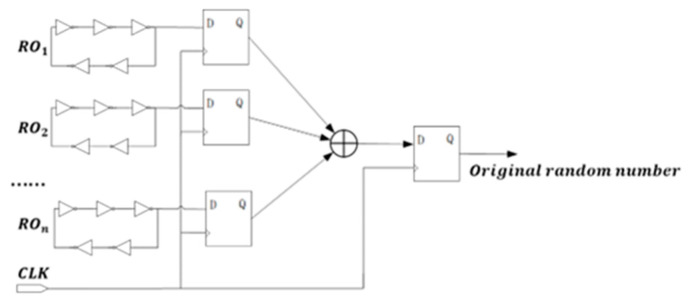
Circuit structure of the true random number generator based on multi-ring oscillators.

The basic architecture of a random number generator based on multi-ring oscillators is illustrated in Figure 2. This configuration comprises two main components: the entropy source and the entropy extraction circuit. The entropy source consists of multiple ring oscillators, each producing a high-frequency oscillatory signal. These signals are sampled by D flip-flops to generate a low-frequency signal, which is subsequently processed through a multi-way XOR operation. The final output signal is then sampled by a D flip-flop to produce the raw random number.

The ring oscillator consists of an odd number of inverters, and during operation, factors such as channel thermal noise and flicker noise in transistors lead to random timing errors in the inverters. These errors manifest as timing jitters in the output of the oscillator, which follows a Gaussian distribution. To achieve sufficient entropy, a parallel multi-ring structure is employed.

**Figure 3:**
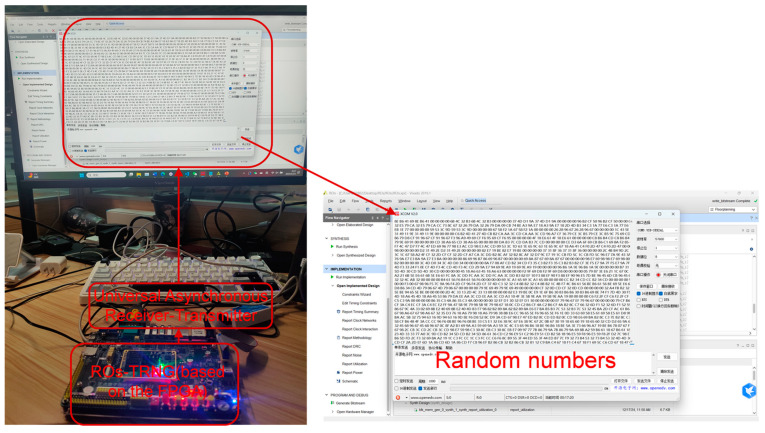
Schematic of Ros-TRNG Built on FPGA Development Board.

As shown in Figure 3, FPGA is used to implement random numbers with different ring counts, specifically using the Zynq-7020 platform. The oscillator ring is constructed using five-stage inverters, with an oscillation frequency of approximately 200 MHz. Sampling is performed using a 10 MHz clock signal, and the outputs are combined through multi-way XOR to produce a single output. To simulate the variation in the quality of random numbers in practical applications of multi-ring oscillators, the number of oscillation rings is set to K={8,12,16,20,24,28,32,64,128}. For a fixed ring count of 32, different sampling frequencies $f_{sample}=\left\{10,20,30,40,50,60,70,80\right\}\;MHz$ are set.

#### 2.2.2. LCG-DRNG Setup

To evaluate the robustness of the neural network prediction model, this paper introduces a pseudo-random number generator based on the Linear Congruential Generator (LCG), a widely used algorithm known for its simplicity and ease of implementation. The LCG generates a series of pseudo-random numbers using a recursive formula. Its mathematical expression is as follows:

(2) $$X_{n+1}=(aX_{n}+c)modM$$

Here, $X=\{X_{0}{,X}_{1}{,X}_{2},X_{3}{,\ldots ,X}_{n}\}$ represents the random number sequence, with $X_{0}$ being the initial value ($0\leq X_{0}<M$). The initial value, or seed, determines the starting point of the random number sequence, with different seed values generating different sequences. M is the modulus of the generator, typically chosen to be a large number to achieve a longer random number cycle, commonly a large prime or a power of 2 ($2^{k}$). a is the multiplier of the generator, typically chosen such that a and M are coprime while c is the increment, and it must also be coprime with M.

When the parameters are correctly chosen, the period of the random sequence equals M for any seed. In our experiment, we selected a={25214903917}, c=1, and $M=\{2^{24},2^{28},2^{32},2^{36}\}$. Random numbers were generated using Python 3.8. To obtain sequences with varying degrees of randomness, pseudo-random sequences with different periods were generated.

### 2.3. Evaluation of Different Datasets

This paper evaluates the performance of LCG pseudo-random numbers with different periods and random numbers generated by a ring oscillator (RO) implemented on FPGA using the NIST-STS statistical suite and the NIST-90B minimum entropy test.

The NIST SP 800-90B statistical test suite employs five independent statistical tests to calculate the minimum entropy, including the Collision, Partial Collection, Markov, Compression, and Frequency tests [32]. In 2018, NIST formally adopted four predictors—MultiMCW, Lag, MultiMMC, LZ78Y, and MultiMA—to improve the accuracy of minimum entropy prediction [33]. Minimum entropy serves as a measure of the randomness of numbers, with higher entropy values indicating that the random numbers are more difficult to predict, thus enhancing the security of the entropy source. Among these tests, the minimum entropy of the source is determined by selecting the smallest value from the results.

The analysis examines the random number performance under varying ring counts and sampling frequencies. Table 1, Table 2 and Table 3 present the results of the NIST suite tests and minimum entropy values for each dataset.

As shown in Table 1, when the period of the LC-RNG exceeds $2^{32}$, the generated pseudo-random numbers successfully pass all NIST statistical tests. Moreover, the statistical *p*-values differ from those obtained when the period is $2^{36}$, suggesting the potential presence of intrinsic correlations within the generated sequences.

The performance of ROs-generated random numbers under different ring counts and sampling frequencies in the NIST statistical suite and minimum entropy tests is presented in Table 2 and Table 3. The results demonstrate that for sampling frequencies below 10 MHz, ROs-RNG with more than 32 rings successfully passes the NIST statistical tests. Additionally, as the number of rings increases, the minimum entropy value also shows a gradual improvement. However, when the ring count is fixed at 32, an increase in sampling frequency leads to a reduction in randomness.

### 2.4. Data Collection and Preprocessing

In the data collection phase, this study utilizes datasets of pseudo-random numbers with varying periods and multi-ring random numbers with different ring counts, each containing 10,000,000 random numbers. These datasets undergo testing with the NIST statistical suite and the minimum entropy test from NIST SP 800-90B, with performance evaluated across four predictors and the final accuracy reported as the lowest value among them. The minimum entropy test is performed with 10 trials for each dataset, and the average result is used. Following the evaluation, 400,000 datasets are selected for training and 500,000 for testing, with the test set divided into 5 groups of 100,000 datasets each to ensure experimental rigor.

**Figure 4:**
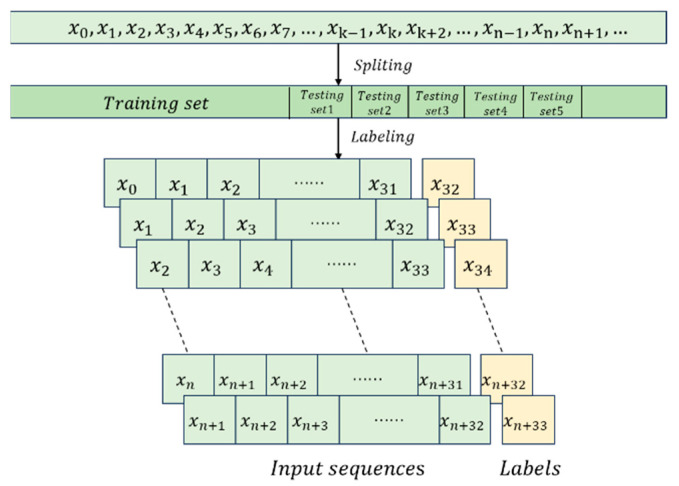
Grouping and Labeling of the Dataset.

The internal correlation of the sequences is determined by evaluating the prediction accuracy of the subsequent bit, which is intrinsically linked to the quality of the random numbers. As shown in Figure 4, the data processing uses 32 consecutive bits as the input sequence, with the 33rd bit serving as the corresponding label. The data are then incrementally shifted by one bit to update the input sequence and label accordingly until all datasets are grouped and labeled. The labeled data are subsequently used for training and testing the neural network.

## 3. Model Design

**Figure 5:**
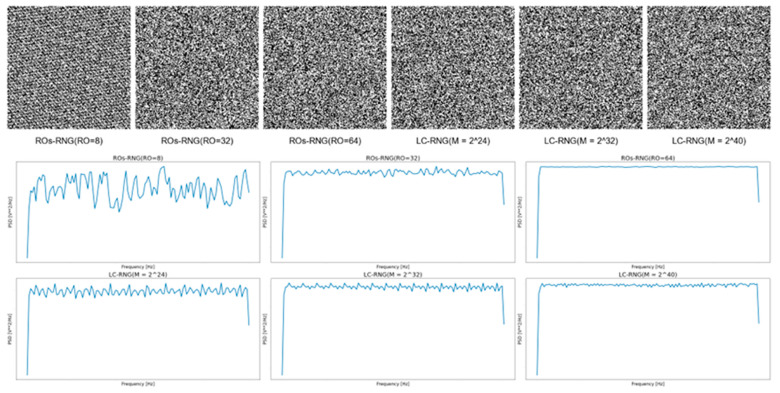
Visualization of Time-Domain and Frequency-Domain for LC-RNG with Different Periods and Ros-RNG with Different Ring Counts.

This study visualizes random numbers with varying degrees of randomness in both the time and frequency domains. In the time domain, the random numbers are represented as QR codes, facilitating the direct observation of the distribution of 0 s and 1 s. In the frequency domain, the power spectral density (PSD) is employed to analyze the frequency characteristics of the random numbers, with the corresponding visualizations provided in Figure 5.

As previously discussed, the randomness of the numbers is closely related to the number of rings and period size: larger numbers of rings and longer periods generally correlate with greater randomness. Time-domain plots reveal that some random number sequences exhibit clear periodic patterns, except for those with notably low entropy. In contrast, sequences with higher randomness show no discernible structure, with the 0 s and 1 s appearing uniformly and randomly distributed.

In the frequency domain, ideal random numbers should exhibit a flat PSD curve. The results demonstrate that data with poor randomness display substantial fluctuations in the PSD plot, whereas data with stronger randomness tend to produce flatter PSD curves. Consequently, in the development of correlation identification models, the focus should be placed on frequency-domain features.

### 3.1. Design of the Deep Learning Network Framework

Traditional Recurrent Neural Network (RNN) models often encounter the challenges of gradient vanishing or exploding when processing long sequence data, which limits their ability to learn long-term dependencies [34]. In contrast, LSTM (Long Short-Term Memory network) effectively addresses these issues with its unique gating mechanisms, including forget gates, input gates, and output gates, enabling sustained data dependency and making it highly suitable for long-duration sequence prediction [35]. Additionally, visualizing the power spectral density (PSD) of random numbers reveals rich features hidden within the frequency domain. The Fast Fourier Transform (FFT) efficiently converts time-domain features into frequency-domain features, while the Feedforward Neural Network (FNN), due to its simple structure and ease of training, excels in handling time series signals with pronounced nonlinear characteristics [36].

The frequency-domain characteristics of random numbers provide insights into potential phase relationships and harmonic connections between signals, offering a distinct advantage over time-domain features in revealing periodicity and correlations. However, time-domain features complement this by capturing details that may be overlooked in frequency-domain analysis through direct examination of the time series. The integration of both time-domain and frequency-domain features allows for a more comprehensive exploration of the hidden, non-intuitive correlations between signals. This process is grounded in multimodal learning, where time-domain and frequency-domain modalities offer complementary information that enhances feature representation. By processing both domains through separate deep network branches, each can learn domain-specific features. Fusion of these features enables the network to leverage the unique strengths of both representations, resulting in a more robust and informative feature vector. This process also taps into the deep neural network’s ability to model complex relationships between the time-domain and frequency-domain signals. In tasks such as periodic signal prediction, anomaly detection, or classification, frequency-domain features enhance model performance by providing periodicity information while time-domain data unveil finer sequence variations. Therefore, the combination of these features increases the accuracy and robustness of models when addressing these tasks.

**Figure 6:**
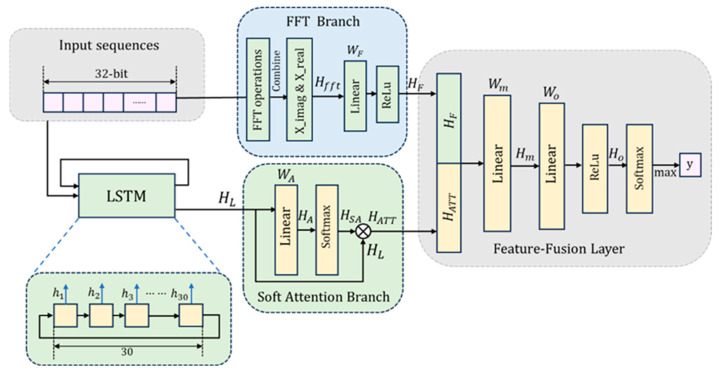
Schematic Diagram of the Deep Learning Architecture Proposed in This Study.

By merging time-domain and frequency-domain features, this study substantially improves the prediction accuracy and efficiency of random number sequences. LSTM processes the time-domain information, while FFT handles the frequency-domain components. The soft attention mechanism works in conjunction with fully connected layers to guide the model’s focus toward the most critical features, thereby optimizing overall prediction performance. The schematic representation is provided in Figure 6.

The input random number sequence is first divided into 32-bit blocks, denoted as $X_{n}$. The time-domain branch processes these sequences through 30 LSTM units, with each unit outputting a hidden state $H_{L}=LSTM\left(X_{n}\right)$ at each corresponding time step. These hidden states are subsequently fed into the soft attention mechanism.

A simplified soft attention layer is employed to weight the input features, emphasizing the most salient information. This layer consists of a fully connected layer followed by a Softmax layer, which maps the input features to a consistent dimensionality. The attention mechanism calculates the weight of each feature, assigning higher weights to important features and lower weights to relatively less important ones. This allows the model to establish relationships between different parts of the input and perform weighted contributions from different features. Specifically, given the input tensor $H_{L}$, attention scores $H_{A}\;$ are computed through a linear transformation:

(3) $$H_{A}={Liner(X)=W}_{A}*H_{L}+B_{A}$$

Softmax is applied to normalize these scores along the feature dimension, yielding the relative importance of each feature for the current sample:

(4) $$H_{SA}=Softmax(H_{A})$$

After normalization, the attention weights are element-wise multiplied with the original input features to generate the weighted feature representation:

(5) $$H_{ATT}=H_{SA}*X$$

The output tensor $H_{ATT}\;$ is reshaped to match the original input dimensions, preserving the structure. This mechanism enables the model to focus on the most relevant parts of the input.

The frequency-domain branch transforms the time-domain signal  X  into its frequency-domain representation using Fast Fourier Transform (FFT), yielding real and imaginary components. These features can reveal the periodicity and correlations within the sequence. By analyzing the frequency-domain signals, the model can identify the frequency components of the signal, effectively capturing the underlying patterns within the sequence. These components are then processed through a fully connected layer with 16 neurons and an ReLU activation to extract frequency-related features:

(6) $$H_{F}=ReLu\left({W_{F}*H}_{fft}+B_{F}\right)\;\;\;\;{[H}_{fft}=FFT\left(X\right)=\{X_{real}{,X}_{imag}\}]$$

In the feature fusion stage, the outputs from the time-domain and frequency-domain branches are concatenated and passed through a fully connected layer with 30 neurons:

(7) $$H_{m}=Concatenate\left(H_{F},H_{ATT}\right)=W_{m}*\left\{H_{F},H_{ATT}\right\}+B_{m}$$

These fused features are further processed through a fully connected layer with 20 neurons and an ReLU activation:

(8) $$H_{o}=ReLu(W_{o}*H_{m}+B_{o})$$

Finally, the model outputs the predicted class through a Softmax layer:

(9) $$\hat{y}=Softmax\left(H_{o}\right)$$

This architecture significantly enhances the prediction accuracy of random number sequences by integrating both time-domain and frequency-domain features. LSTM processes the time-domain information, capturing the instantaneous changes within the sequence, while FFT handles the frequency-domain components, revealing the periodic elements of the sequence. The soft attention mechanism, combined with fully connected layers, ensures that the model focuses on the most critical features, thereby optimizing overall predictive performance. By fusing these two types of features, the model can identify non-intuitive correlations within the sequence.

### 3.2. System Evaluation

In this study, the neural network’s prediction accuracy is employed to evaluate the presence of correlation in random sequences. In an ideal and unpredictable random system, the probabilities of 0 and 1 are equal, with each occurring at a 50% rate. Consequently, a minimum entropy approaching 1 indicates a higher degree of randomness in the system. The neural network’s prediction accuracy is calculated as:

(10) $$P_{pre}=\frac{N_{T}}{N_{T}+N_{F}}\times 100\%$$

where $N_{T}$ is the number of successful predictions for the next bit, and $N_{F}$ is the number of failed predictions.

For an ideal random number sequence, the assumption of independence and identical distribution holds, indicating that each bit is independent and there is no correlation between them. According to the central limit theorem [37], the prediction for the next bit follows a distribution $X\sim N(0.5,\frac{1}{2\sqrt{n}})$, where n is the number of predictions. In the experiments, the test set contains 100,000 sequences, which leads to n=100,000 predictions and a standard deviation of σ=0.00158. Set 3σ and 5σ as the boundaries, corresponding to $P_{b1}=0.5+3\sigma =0.50474$ and $P_{b2}=0.5+5\sigma =0.5079$, respectively. If the prediction probability is higher than $P_{b2}$, it indicates that there is a 99.9% probability of identifying the correlation between sequences, which, at this time, means that there is an obvious correlation among the random numbers. If the prediction probability is lower than $P_{b1}$, it indicates that the correlation among the random numbers is extremely small. When the prediction probability is between $P_{b1}\;$ and $P_{b2}$, it indicates that there is a risk of weak correlation among the random numbers.

## 4. Results and Discussion

### 4.1. Overall Workflow

The experimental procedure in this study is structured into three primary phases: data collection and preprocessing, model training and validation, and result evaluation and analysis. Initially, in the data collection and preprocessing phase, random numbers with varying degrees of randomness are collected, categorized, and labeled accordingly; these processed data are then split into training and testing datasets. In the subsequent model training and validation phase, the preprocessed data are input into the configured neural networks for training and validation with prediction accuracies recorded for performance evaluation. Finally, in the result evaluation and analysis phase, the randomness of the generated random numbers under different conditions is assessed using statistical suites, with the resulting evaluations correlated with the prediction accuracies of the neural networks to explore the relationship between prediction accuracy and entropy values.

### 4.2. Presentation of Experimental Details

In this study, the random number prediction task is formulated as a multi-class classification problem, with the cross-entropy loss function employed to quantify the discrepancy between the model’s predictions and the true labels. The Adam optimizer is utilized to minimize this loss function, guiding the model’s parameter updates throughout the training process.

To assess the efficacy of the proposed model, it is benchmarked against five baseline networks: FNN, RNN, LSTM, TCN, and Transformer. The specific parameter configurations of these baseline networks are detailed in Table 4. Notably, the Residual Blocks in the model consist of three layers, each comprising two convolutional operations, with the dilation factor for each convolutional layer set to 2. The dilation factor increases with the depth of the layer, where the first layer has a dilation factor of 1, the second layer has a factor of 2, and the third layer has a factor of 4. The Embedding Layer maps the raw sequence data into 16-dimensional vectors, incorporating positional encoding to ensure the model captures the time-step positional information. The Transformer Encoder Layer processes the input data using self-attention mechanisms and feed-forward networks to produce 16-dimensional output vectors at each layer, and the final output after two layers of processing is used for the classification task.

### 4.3. Analysis and Discussion of Experimental Results

This paper provides a quantitative evaluation of the prediction performance of various neural network models for random number generation, focusing on the accuracy of their predictions. The experimental results reveal that the FFT-ATT-LSTM model consistently outperforms other networks in prediction accuracy across different periods. As shown in Table 5, for a period of M = $2^{24}$, the FFT-ATT-LSTM model achieves a prediction accuracy of 71.42%, substantially higher than the LSTM model at 66.96% and the FNN model at 60.92%, showing improvements of 4.46 percentage points and 10.5 percentage points, respectively. It is noteworthy that when the period is extended to M = $2^{32}$ and the random numbers pass the NIST statistical suite tests, the prediction accuracy of the proposed model exceeds the threshold $P_{b2}$, indicating the model’s capability to identify internal correlations within random numbers. This demonstrates the model’s enhanced ability to handle long sequences and detect correlations within random number sequences through the integration of FFT and attention mechanisms.

In the case of the ROs-RNG model, when the number of rings exceeds 32, the random numbers pass the NIST statistical suite tests. As shown in Table 6, the prediction accuracy of the FFT-ATT-LSTM model consistently outperforms the other baseline networks, particularly when the number of rings is set to 32. Under the condition that the random numbers pass the NIST statistical suite tests, the prediction accuracy of the FFT-ATT-LSTM model also exceeds the threshold, while the accuracy of all other baseline models falls short of this threshold. This demonstrates the model’s superior ability to identify internal correlations within random numbers. This further validates the effectiveness of combining FFT with attention mechanisms to uncover hidden correlations within random number sequences, thereby enhancing prediction performance.

**Figure 7:**
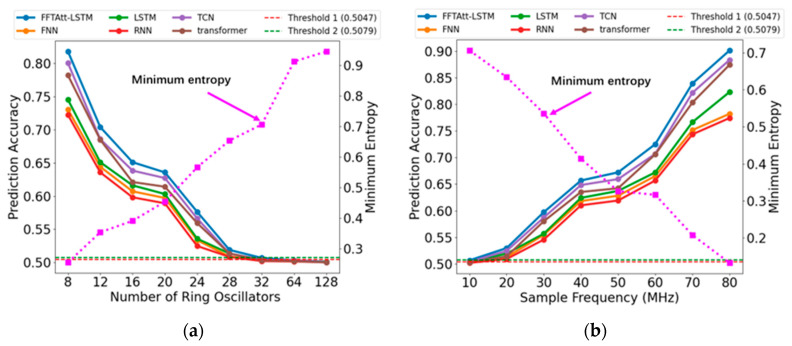
Comparison of Prediction Performance and Minimum Entropy Values for Different Neural Network Models under Varying Conditions of Random Sequences: (**a**) Evaluation Results for Random Numbers Based on Different Ring Counts (8–128); (**b**) Evaluation Results for Random Numbers Based on Different Sampling Frequencies (10–80 MHz).

Figure 7 illustrates the prediction performance of various neural network models under different conditions of ring counts and sampling frequencies. The random numbers generated by the multi-ring random number generator exhibit varying degrees of randomness, with the sampling frequency influencing their randomness. Specifically, an increase in the number of rings leads to a rise in the minimum entropy of the random numbers, while an elevation in the sampling frequency results in a decrease in minimum entropy. Among the six evaluated neural networks, the traditional RNN shows the poorest performance due to challenges such as gradient explosion and vanishing gradients.

In contrast, the incorporation of memory gates and other mechanisms in the LSTM model significantly enhances its ability to predict time series, allowing it to outperform both the FNN and RNN models. Transformer and TCN process sequential data in distinct manners: Transformer leverages self-attention mechanisms to capture long-range dependencies, while TCN utilizes 1D convolutions to model local dependencies. Under the constraints of limited sequence length and smaller datasets, the FFT-ATT-LSTM model proposed in this paper delivers superior performance, achieving a prediction accuracy of 90.12%, which exceeds that of TCN (88.34%), Transformer (87.43%), FNN (78.21%), RNN (77.42%), and LSTM (82.31%) by approximately 8 percentage points.

To further substantiate the role of the FFT layer in identifying inherent randomness and periodic patterns within the random numbers, an ablation experiment was performed. The results presented in Table 7 show that for datasets with substantial fluctuations in the PSD plot, removing the FFT layer leads to a reduction in prediction accuracy by approximately 8–9%. This indicates that the FFT layer effectively extracts information from the PSD fluctuations, thereby enhancing prediction accuracy. For datasets exhibiting relatively flat Power Spectral Density (PSD) plots, the prediction accuracy of the ablated network is virtually identical to that of the original network, indicating that the LSTM’s ability to process time-domain information becomes the predominant factor in achieving accurate predictions. In contrast, for datasets with a period of $M=2^{32}$, the network incorporating the FFT layer maintains superior performance, not only surpassing the accuracy of the ablated network but also consistently exceeding the threshold (50.79%). This further substantiates the claim that the fusion of time-domain and frequency-domain features enhances the model’s capability to detect subtle correlations within random number sequences.

Table 8 summarizes the training and inference times, along with the hardware resource consumption of various models. The FNN and RNN models exhibit relatively small parameter sizes (6.45 KB and 7.74 KB, respectively) and lower computational complexity. However, their ability to identify correlations in random number sequences is limited, with prediction accuracies falling below the threshold under certain conditions, all of which are inferior to the model proposed in this study. In contrast, the TCN and Transformer models, with larger parameter sizes (41.51 KB and 61.51 KB, respectively) and higher computational complexity, demonstrate superior prediction accuracy compared to FNN, RNN, and LSTM. However, their capacity to detect correlations remains suboptimal relative to the FFT-ATT-LSTM model. The FFT-ATT-LSTM model, with a parameter size similar to that of LSTM (approximately 30 KB), surpasses LSTM in prediction accuracy by approximately 8 percentage points. Furthermore, it not only exceeds the accuracy threshold but also successfully passes the NIST statistical suite tests, thereby demonstrating its effectiveness in identifying subtle correlations within random number sequences.

**Figure 8:**
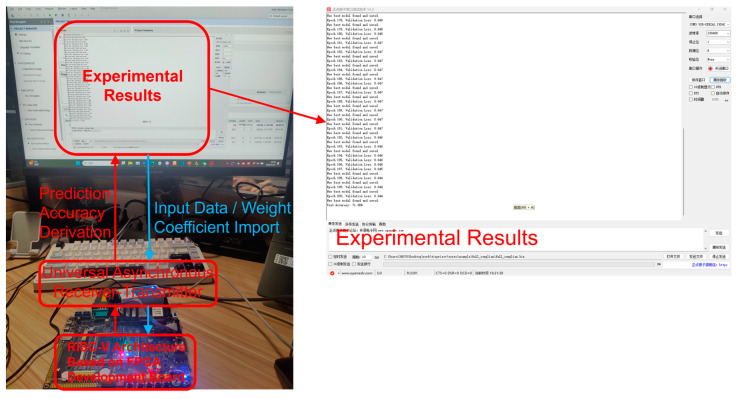
Schematic Diagram of Hardware Implementation.

This paper implements the proposed algorithm on an edge device, with the experimental platform built on a RISC-V microprocessor system based on the Zynq-7020 development board. The hardware resource consumption is summarized in Table 9. The network model parameters and computation methods are compiled using C language and loaded onto the RISC-V processor, enabling online training and forward inference evaluation of random numbers. The experimental process is illustrated in Figure 8, where data are imported into the development board via the UART serial port. The board performs on-chip weight updates for both the training and test datasets. Finally, the training results are transmitted back to the PC via UART for prediction accuracy observation. This paper presents prediction experiments for the LC-RNG and ROs-RNG under two different randomness conditions. The experimental results are shown in Table 10. Although the on-chip training time is relatively long, approximately 30 min, the prediction accuracy is consistent with the PC-based results. This result preliminarily validates the feasibility of implementing the algorithm on edge devices. Future research will focus on optimizing the neural network model and incorporating hardware accelerators to enhance computational efficiency.

## 5. Conclusions

This paper introduces an innovative deep learning architecture, Fast Fourier Transform-Attention Mechanism-Long Short-Term Memory Network (FFT-ATT-LSTM), designed to enhance the identification of hidden correlations within random number sequences by effectively integrating both time-domain and frequency-domain features. Experimental results demonstrate a significant negative correlation between minimum entropy values and the network’s prediction accuracy, with a Pearson correlation coefficient of −0.925 and a *p*-value of 1.07 × 10^−7^. This finding offers a novel approach for evaluating entropy source quality. The model incorporates a simplified soft attention mechanism that enhances the Long Short-Term Memory (LSTM) network’s ability to capture time-domain features. Meanwhile, the Fast Fourier Transform (FFT) extracts frequency-domain features, enabling the effective fusion of multi-modal characteristics. Experimental outcomes reveal that when predicting random numbers generated by linear congruential generators and multi-ring random number generators, FFT-ATT-LSTM improves prediction accuracy by 4.46% and 8%, respectively, compared to baseline networks. Ablation studies on the FFT layer further confirm the significance of frequency-domain features, as its removal results in an 8–9% reduction in prediction accuracy. Moreover, FFT-ATT-LSTM maintains its ability to detect correlations within sequences (above the threshold), even when the prediction accuracy of other baseline networks falls below the threshold. The FFT-ATT-LSTM network exhibits a hardware overhead of 33.90 KB, which is higher than that of traditional networks such as FNN, RNN, and LSTM. However, it significantly outperforms these models in terms of prediction accuracy. It also surpasses models such as TCN (41.51 KB) and Transformer (61.51 KB), both in terms of model size and performance. Given its optimal balance between hardware resource requirements and prediction performance, FFT-ATT-LSTM offers a superior cost-performance ratio. The paper also presents an embedded implementation of the model, demonstrating the feasibility of its online deployment. Future work will focus on further investigating the relationship between prediction accuracy and minimum entropy values, with the aim of exploring the feasibility of inferring minimum entropy from prediction accuracy. Additionally, the network’s architecture will be further optimized to enhance its performance in real-time health monitoring of entropy sources in random number generators.

## Figures and Tables

**Table 1:** Results of NIST Statistical Test Suite and NIST 90B Minimum Entropy on LC-RNG Datasets at Different Stages.

Statistical Tests	LC-RNG							
	$M=2^{24}$		$M=2^{28}$		$M=2^{32}$		$M=2^{36}$	
	*p*-Value	Result	*p*-Value	Result	*p*-Value	Result	*p*-Value	Result
Frequency	0.321384	Success	0.424134	Success	0.534146	Success	0.668821	Success
Block Frequency	0.157892	Success	0.324134	Success	0.532132	Success	0.660658	Success
Cumulative Sums	0.013142	Success	0.036413	Success	0.122325	Success	0.002043	Success
Runs	0.731543	Success	0.863452	Success	0.911413	Success	0.066822	Success
Longest Run	0.324141	Success	0.431241	Success	0.534146	Success	0.739918	Success
Rank	0.531134	Success	0.778635	Success	0.911413	Success	0.723103	Success
FFT	0.000000	Failure	0.234131	Success	0.593681	Success	0.327831	Success
Non-overlapping Template	0.000000	Failure	0.000000	Failure	0.350485	Success	0.534146	Success
Overlapping Template	0.006215	Success	0.112378	Success	0.234351	Success	0.350485	Success
Universal	0.000000	Failure	0.098321	Success	0.122325	Success	0.347842	Success
Approximate Entropy	0.000000	Failure	0.934134	Success	0.997721	Success	0.999920	Success
Random Excursions	0.432421	Success	0.524141	Success	0.735211	Success	0.987277	Success
Random Excursions Variant	0.283741	Success	0.413673	Success	0.507518	Success	0.887214	Success
Serial	0.604215	Success	0.735213	Success	0.969284	Success	0.987277	Success
Linear Complexity	0.450524	Success	0.633256	Success	0.911413	Success	0.940102	Success
Total successful tests	11/15		14/15		15/15		15/15	
NIST-90B (Minimum entropy)	0.4578		0.6753		0.6962		0.7246	

**Table 2:** Results of NIST Statistical Test Suite and NIST 90B Minimum Entropy on ROs-RNG Datasets with Different Ring Counts.

Statistical Tests	ROs-RNG								
	RO=8	RO=12	RO=16	RO=20	RO=24	RO=28	RO=32	RO=64	RO=128
	Result	Result	Result	Result	Result	Result	Result	Result	Result
Frequency	Success	Success	Success	Success	Success	Success	Success	Success	Success
Block Frequency	Failure	Success	Success	Success	Success	Success	Success	Success	Success
Cumulative Sums	Failure	Failure	Failure	Failure	Success	Success	Success	Success	Success
Runs	Failure	Success	Success	Success	Success	Success	Success	Success	Success
Longest Run	Success	Success	Success	Success	Success	Success	Success	Success	Success
Rank	Success	Success	Success	Success	Success	Success	Success	Success	Success
FFT	Failure	Failure	Failure	Failure	Failure	Failure	Success	Success	Success
Non-overlapping Template	Failure	Failure	Failure	Failure	Failure	Success	Success	Success	Success
Overlapping Template	Failure	Failure	Success	Success	Success	Success	Success	Success	Success
Universal	Failure	Failure	Failure	Failure	Success	Success	Success	Success	Success
Approximate Entropy	Failure	Failure	Failure	Failure	Failure	Success	Success	Success	Success
Random Excursions	Success	Success	Success	Success	Success	Success	Success	Success	Success
Random Excursions Variant	Success	Success	Success	Success	Success	Success	Success	Success	Success
Serial	Failure	Failure	Failure	Failure	Success	Success	Success	Success	Success
Linear Complexity	Success	Success	Success	Success	Success	Success	Success	Success	Success
Total successful tests	6/15	8/15	9/15	9/15	12/15	14/15	15/15	15/15	15/15
NIST-90B (Minimum entropy)	0.2553	0.3538	0.3912	0.4538	0.5663	0.6538	0.7063	0.9124	0.9452

**Table 3:** Results of NIST Statistical Test Suite and NIST 90B Minimum Entropy on ROs-RNG Datasets with Different Sampling Frequencies.

Statistical Tests	ROs-RNG (RO = 32)						
	10 MHz	20 MHz	30 MHz	40 MHz	50 MHz	60 MHz	70 MHz
	Result	Result	Result	Result	Result	Result	Result
Frequency	Success	Success	Success	Success	Success	Success	Success
Block Frequency	Success	Success	Success	Failure	Failure	Failure	Failure
Cumulative Sums	Success	Success	Success	Success	Success	Failure	Failure
Runs	Success	Success	Success	Success	Success	Failure	Failure
Longest Run	Success	Success	Success	Success	Success	Success	Success
Rank	Success	Success	Success	Success	Success	Success	Success
FFT	Success	Failure	Failure	Failure	Failure	Failure	Failure
Non-overlapping Template	Success	Success	Failure	Failure	Failure	Failure	Failure
Overlapping Template	Success	Success	Success	Success	Success	Success	Failure
Universal	Success	Success	Success	Failure	Failure	Failure	Failure
Approximate Entropy	Success	Success	Success	Success	Failure	Failure	Failure
Random Excursions	Success	Success	Success	Success	Success	Success	Failure
Random Excursions Variant	Success	Success	Success	Success	Success	Success	Success
Serial	Success	Success	Success	Failure	Failure	Failure	Failure
Linear Complexity	Success	Success	Success	Success	Success	Success	Success
Total successful tests	15/15	14/15	13/15	10/15	9/15	7/15	5/15
NIST-90B (Minimum entropy)	0.7063	0.6341	0.53625	0.4137	0.3262	0.3162	0.2075

**Table 4:** Model configurations of different baseline networks.

FNN-Based Model	RNN-Based Model	LSTM-Based Model	TCN-Based Model	Transformer-Based Model
Input layer	Input layer	Input layer	Input layer	Input layer
FC-30 + Relu	RNN-30 + Relu	LSTM-30 + Relu	Residual Blocks1	Embedding Layer-16 + Positional Encoding (Tanh)
FC-20 + Relu	FC-2 + Softmax	FC-2 + Softmax	Residual Blocks2	Transformer Encoder Layers1 (nhead = 4)
FC-2 + Softmax	/	/	Residual Blocks3	Transformer Encoder Layers2 (nhead = 4)
/	/	/	FC-16+ Relu	FC-64
/	/	/	FC-2+Softmax	FC-2 + Softmax

**Table 5:** Prediction Performance of Different Models for LC-RNG with Varying Periods.

Model	LC-RNG (Accuracy: %)			
	$M=2^{24}$	$M=2^{28}$	$M=2^{32}$	$M=2^{36}$
FNN-Based Model	60.92±0.02	54.45±0.01	50.33±0.03	50.29±0.01
RNN-Based Model	54.15±0.02	51.79±0.04	50.15±0.02	50.05±0.02
LSTM-Based Model	66.96±0.02	59.84±0.02	50.41±0.02	50.32±0.02
TCN-Based Model	58.18±0.02	56.97±0.03	50.26±0.02	50.43±0.02
Transformer-Based Model	52.35±0.02	51.26±0.07	50.03±0.02	49.04±0.02
FFT-ATT-LSTM-Based Model	71.42±0.01	68.52±0.04	50.81±0.02	50.48±0.03

**Table 6:** Prediction Performance of Different Models for ROs-RNG with Varying Ring Counts.

Model	ROs-RNG (Accuracy: %)			
	RO=28	RO=32	RO=64	RO=128
FNN-Based Model	50.89±0.02	50.32±0.01	50.14±0.03	50.08±0.01
RNN-Based Model	50.84±0.05	50.21±0.04	50.15±0.02	50.02±0.01
LSTM-Based Model	51.21±0.01	50.41±0.02	50.21±0.02	50.05±0.02
TCN-Based Model	51.35±0.03	50.42±0.03	50.32±0.02	50.01±0.03
Transformer-Based Model	51.33±0.02	50.32±0.07	50.21±0.02	50.02±0.02
FFT-ATT-LSTM-Based Model	51.89±0.02	50.68±0.01	50.32±0.02	50.12±0.03

**Table 7:** Ablation Study Results for the FFT Layer of FFT-ATT-LSTM.

Model	ROs-RNG (Accuracy: %)		LC-RNG (Accuracy: %)	
	RO=8	RO=64	$M=2^{32}$	$M=2^{36}$
ATT-LSTM-Based Model	73.54±0.02	50.22±0.02	62.46±0.02	50.41±0.02
FFT-ATT-LSTM-Based Model	81.81±0.02	50.32±0.02	71.42±0.01	50.81±0.02

**Table 8:** Comparison of Training and Inference Times, and Hardware Resource Consumption for Different Models.

Model	Training Time per Epoch (s)	Inference Time (s)	Params	Flops
FNN-Based Model	7.12	3.06	6.45KB	1600
RNN-Based Model	3.51	2.19	7.74KB	2010
LSTM-Based Model	6.31	2.67	30.24KB	7980
TCN-Based Model	5.62	4.74	41.51KB	332,320
Transformer-Based Model	4.95	3.51	61.51KB	479,072
FFT-ATT-LSTM-Based Model	6.56	2.82	33.90 KB	254,400

**Table 9:** Hardware Resource Usage.

Resource	Utilization	Available	Utilization Rate (%)
LUT	31,498	203,800	15.46
LUTRAM	16,699	64,000	26.09
FF	19,326	407,600	4.74
BRAM	44.50	445	10
DSP	62	840	7.38
BUFG	5	32	10

**Table 10:** The prediction accuracy and time of the FFT-ATT-LSTM network for on-chip training of random numbers with different randomness.

Dataset	Prediction Accuracy (%)	On-Chip Training Prediction Accuracy (%)	On-Chip Training Time (min)
LC-RNG ($M=2^{24}$)	71.42	71.38	33.8 min
LC-RNG ($M=2^{36}$)	50.48	50.39	34.7 min
ROs-RNG (RO=28)	51.89	51.71	33.4 min
ROs-RNG (RO=64)	50.32	50.21	34.3 min

## Data Availability

The data that support the findings of this study are available from the corresponding author upon reasonable request.
